# Upregulation of IRF9 Contributes to Pulmonary Artery Smooth Muscle Cell Proliferation During Pulmonary Arterial Hypertension

**DOI:** 10.3389/fphar.2021.773235

**Published:** 2021-12-01

**Authors:** Yong-Jie Chen, Yi Li, Xian Guo, Bo Huo, Yue Chen, Yi He, Rui Xiao, Xue-Hai Zhu, Ding-Sheng Jiang, Xiang Wei

**Affiliations:** ^1^ Division of Cardiothoracic and Vascular Surgery, Sino-Swiss Heart-Lung Transplantation Institute, Tongji Hospital, Tongji Medical College, Huazhong University of Science and Technology, Wuhan, China; ^2^ Department of Cardiovascular Surgery, Union Hospital, Fujian Medical University, Fuzhou, China; ^3^ Department of Pathophysiology, School of Basic Medicine, Tongji Medical College, Huazhong University of Science and Technology, Wuhan, China; ^4^ Key Laboratory of Pulmonary Diseases of Ministry of Health, Tongji Medical College, Huazhong University of Science and Technology, Wuhan, China; ^5^ Key Laboratory of Organ Transplantation, Ministry of Education; NHC Key Laboratory of Organ Transplantation, Key Laboratory of Organ Transplantation, Chinese Academy of Medical Sciences, Wuhan, China

**Keywords:** pulmonary arterial hypertension, pulmonary artery smooth muscle cell, interferon regulator factor 9, mitochondrial function, Akt, Phb1

## Abstract

Abnormal proliferation of pulmonary artery smooth muscle cells (PASMCs) is a critical pathological feature in the pathogenesis of pulmonary arterial hypertension (PAH), but the regulatory mechanisms remain largely unknown. Herein, we demonstrated that interferon regulatory factor 9 (IRF9) accelerated PASMCs proliferation by regulating Prohibitin 1 (PHB1) expression and the AKT-GSK3β signaling pathway. Compared with control groups, the rats treated with chronic hypoxia (CH), monocrotaline (MCT) or sugen5416 combined with chronic hypoxia (SuHx), and mice challenged with CH had significantly thickened pulmonary arterioles and hyperproliferative PASMCs. More importantly, the protein level of IRF9 was found to be elevated in the thickened medial wall of the pulmonary arterioles in all of these PAH models. Notably, overexpression of IRF9 significantly promoted the proliferation of rat and human PASMCs, as evidenced by increased cell counts, EdU-positive cells and upregulated biomarkers of cell proliferation. In contrast, knockdown of IRF9 suppressed the proliferation of rat and human PASMCs. Mechanistically, IRF9 directly restrained PHB1 expression and interacted with AKT to inhibit the phosphorylation of AKT at thr308 site, which finally led to mitochondrial dysfunction and PASMC proliferation. Unsurprisingly, MK2206, a specific inhibitor of AKT, partially reversed the PASMC proliferation inhibited by IRF9 knockdown. Thus, our results suggested that elevation of IRF9 facilitates PASMC proliferation by regulating PHB1 expression and AKT signaling pathway to affect mitochondrial function during the development of PAH, which indicated that targeting IRF9 may serve as a novel strategy to delay the pathological progression of PAH.

## Introduction

Pulmonary arterial hypertension (PAH) is a progressive and devastating lung disease that is precipitated by pulmonary arteriole remodeling, ultimately leading to pulmonary vascular resistance and right heart failure (RHF) (Leopold and Maron, 2016). The incidence of PAH ranges from 2.0 to 7.6 cases per million adults per year (Thenappan et al., 2018). Data from American National Institutes of Health (NIH) suggested that the median survival of patients is now 6 years compared with 2.8 years in the 1980s (Thenappan et al., 2010), and the one-year survival rate of PAH patients is 86%, compared with 65% in the 1990s (Rich et al., 1987; Benza et al., 2010). However, even with these recent advances in survival, available treatment approaches are still currently limited, and for the terminal stage of PAH, lung transplantation is the only choice. Therefore, it is urgent to develop a method of molecular targeting therapy that can reverse the pathogenesis of PAH.

PAH is a vasculopathy that is histopathologically characterized by excessive pulmonary vasoconstriction, abnormal arteriole remodeling and plexiform lesions (Schermuly et al., 2011). In these pathological processes, the cellular phenotype of pulmonary artery smooth muscle cells (PASMCs) changes from a quiescent state to a hyperproliferative state (Leopold and Maron, 2016), and the cancer-like phenotype and metabolic shift are closely aligned with the “Warburg effect” (Vander Heiden et al., 2009). In these cells, mitochondrial glucose oxidation is suppressed, whereas glycolysis is utilized as the major source of adenosine triphosphate production (J. Dai et al., 2018). The PI3K/AKT pathway is a main regulator of cell growth and glucose metabolism, the downregulation of which attenuates glucose uptake and utilization in mitochondria so that more biomass and carbon resources are maintained for the rapid biosynthesis that is essential for cell proliferation (Vander Heiden et al., 2009). Therefore, investigating the molecular mechanism of PASMC proliferation during PAH will provide novel insight into the development of medical therapies for PAH.

Growing evidences indicate that interferon therapy may trigger PAH (Al-Zahrani et al., 2003; Jochmann et al., 2005; Ledinek et al., 2009; Dhillon et al., 2010; Savale et al., 2014). Interferon beta (IFN-β) and interferon alpha (IFN-α) were listed among the substances with a possible risk of PAH induction in the guidelines of the European Society of Cardiology and the European Respiratory Society in 2015 (Galiè et al., 2015). The expression of interferons is regulated by interferon regulator factors (IRFs), and our previous results demonstrated that IRFs play critical roles in cardiovascular diseases (Jiang et al., 2013; Jiang et al., 2014a; Zhang et al., 2014a; Jiang et al., 2014b; Jiang et al., 2014c; Jiang et al., 2014d; Zhang et al., 2015). Moreover, we reported that IRF9 promotes VSMC proliferation and neointima formation induced by carotid wire injury (Zhang et al., 2014b). Although both neointima formation and PAH are related to the excessive proliferation of SMCs, the molecular mechanisms regulating neointima formation and PAH are quite different. Thus, it is very interesting to clarify the roles and mechanisms of IRF9 in PAH.

In the present study, we found that the expression level of IRF9 was elevated in the pulmonary arterioles of chronic hypoxia (CH)- induced rats or mice, and also in monocrotaline (MCT)- or sugen5416 combined with chronic hypoxia (SuHx)- induced rats. Overexpression of IRF9 promoted, while IRF9 knockdown inhibited the proliferation of rat PASMCs (RPASMCs) and human PASMCs (HPASMCs). IRF9 achieved these effects by suppressing the expression of PHB1 and AKT signaling pathway. Therefore, our findings reveal a new pathogenesis of PAH and provide a novel target for the treatment of PAH.

## Materials and Methods

### Pulmonary Arterial Hypertension Models

In the present study, the chronic hypoxia-induced PAH animal models were established in male C57BL/6 mice (weighing 20–25 g) and male Sprague-Dawley rats (weighing 180–200 g) as our previous studies descripted (M. Dai et al., 2019; X. Zeng et al., 2017). In brief, for mice PAH models, they were housed in a chamber and exposed to mixed air with 10% O_2_, 1% CO_2_, and 89% N_2_. At 1, 2, 3 or 4 weeks after hypoxia, the mice were sacrificed for the following detection. The control mice were raised in the chamber with air ventilation. The similar procedures were performed to generate the hypoxia-induced PAH model in rats. As for MCT- and SuHx-induced PAH models were generated as we previously reported in rats (Lu et al., 2015; Xiao et al., 2017). Rats were received once subcutaneous administration of MCT at 60 mg/kg per day, the standard dosage in rodent to produce the PAH model. In the SuHx-induced PAH model, rats were injected subcutaneously with 20 mg/ml sugen5416 (HY-10374, MCE; dissolved in 0.5% carboxymethylcellulose, 0.9% NaCl, 0.4% polysorbate, and 0.9% benzyl alcohol in deionized water) once a week and keep in normobaric hypoxia (10% O_2_ concentration) for consecutive 3 weeks, after that, the rats were moved into normoxia condition for additional 2 weeks. As for rats in control groups, they were injected with saline intraperitoneally and housed in normoxia condition to the end of modeling. All experimental protocols for mice or rats in this study were approved by the Animal Experimental Ethics Committee of the Tongji Hospital, Tongji Medical College, Huazhong University of Science and Technology. The procedures followed the International Association for the Study of Pain guidelines for animal research and standard biosecurity and institutional safety procedures.

### Echocardiography and Hemodynamic Measurements

Right heart function and heart structure are detected by echocardiography. M-mode images derived from the short axis of the right ventricle were recorded using a VisualSonics vevo 1100 imaging system with a 30-MHz probe. The right ventricle internal diameters were obtained from at least three beats and then averaged. After echocardiography experiments, hemodynamic measurements were conducted as described in our previous study (J. Zhang et al., 2012; Zhu et al., 2016). Briefly, after modeling, rats and mice were intraperitoneally administered 3% mebubarbital, and a catheter was inserted in the trachea and connected to room air. A Millar Mikro-Tip catheter (SRP-671, AD Instruments) was set from the right jugular vein into the right heart to collect right ventricular pressure signal and other relative cardiac parameters. By the end of hemodynamic measurements, pre-warmed saline was injected into pulmonary circulation through the pulmonary artery catheter and drained from the left atrium to wash out the blood. Then the lungs and heart tissues were removed and fixed with formaldehyde solution (10%) for 3 days. The right ventricular hypertrophy was quantified by weighting the right ventricular free wall (RV) and the left ventricle (LV) together with the septum (S, LV + S).

### Plasmid Construction

The promoter sequence of human PHB1 gene located between -1,540 and -525 was cloned into the pGL3-basic reporter vector. Full-length human IRF9 and AKT1 CDS sequences were amplified and cloned into the pHAGE-CMV expression vector. Primers of PHB1-promoter: forward 5′-TGC​TAG​CCC​GGG​CTC​GAG​CTC​GTG​TTC​ATG​GAT​TGG​TG-3′; reverse 5′-TAC​CGG​AAT​GCC​AAG​CTT​ACC​AAC​CGA​GAG​GAA​GGA​AT-3′; Primers of IRF9: forward 5′-CCG​ACG​CGT​GCC​ACC​ATG​GCA​TCA​GGC​AGG-3′; reverse 5′- CCG​CTC​GAG​CAC​CAG​GGA​CAG​AAT​G-3′; Primers of AKT1: forward 5′-CCG​ACG​CGT​GCC​ACC​ATG​AGC​GAC​GTG​GCT-3′; reverse 5′-CCG​CTC​GAG​GGC​CGT​GCC​GCT​GGC-3′. Short hairpin RNAs target to human IRF9 and rattus IRF9 were inserted into PLKO.1 vector. The target sequences are as below: human-IRF9-shRNA#1: GCC​ATA​CTC​CAC​AGA​ATC​TTA; rattus-IRF9-shRNA#1: GCA​GAA​CCC​TAC​AAA​GTA​TAT; rattus-IRF9-shRNA#2: GCA​GGC​CTT​TGC​CCG​AAA​TTT.

### Cells Culturing

The isolation and culturing of primary RPASMCs was described in our previous studies (Guo et al., 2019; Chen et al., 2020). Briefly, after anesthesia with 3% chloral hydrate, the pulmonary arteries of the rats were carefully separated from the lungs chilled in PBS solution. After carefully peeling off the adventitia and endarterium of the pulmonary arteries, pulmonary arteries tissue was cut into fragments less than 1 mm in length, placed in a culture flask, and dried for 30 min, after which 5 ml DMEM/F12 (SH30023.01; HyClone) supplemented with 10% fetal bovine serum (FBS; 1767839; Thermo Fisher Scientific) and 1% penicillin-streptomycin (15140-122; Thermo Fisher Scientific). The cells were digested with trypsin and transferred to Petri dishes from culture flasks after the cells were at an appropriate density (approximately 5–7 days) and passaged every two days.

The human pulmonary artery smooth muscle cells (HPASMCs) purchased from Lonza (Catalog #: CC-2581) were culturing in DMEM/F12 supplemented with 10% fetal bovine serum and 1% penicillin-streptomycin as our previous study (Zhu et al., 2021). After transfecting with indicated lentivirus, HPASMCs of hypoxia group were placed in a special cell incubator with 1% O_2_ concentration (cells of normoxia group in incubator with 20% O_2_ concentration) for 48 h. After that, the cells were immediately fixed or lysis for follow-up tests.

### Drug Treatment and Lentivirus Infection

Cells were infected with Lenti-IRF9-Flag, Lenti-Flag, Lenti-PLKO.1, Lenti-shIRF9-1, or Lenti-shIRF9-2 as previously described (Li et al., 2018; Chen et al., 2020). RPASMCs which had infected with indicated lentivirus were seeded at a concentration of 1.2 × 10^5^ in each 6 cm dish or 3 × 10^4^ in each well of 12-well plates. After starvation for 12 h, the second passage of RPASMCs were treated with MK2206 (0.5 μM, S1078; Selleck) for 24 h, and dosing was repeated for another 24 h after renewing the medium. After total 48 h of treatment, RPASMCs were used for growth curve and EdU incorporation assays.

### Immunohistochemistry Staining Analysis

Immunohistochemistry (IHC) staining was performed as described previously (Jiang et al., 2016; Yi et al., 2021). In brief, the paraffin slices were soaked in xylene, 100% ethanol, 95% ethanol and 70% ethanol successively for dewaxing and hydration. After that, the slides were put into EDTA buffer (pH 9.0, MVS-0099, MXB Biotechnologies) and maintained at 100°C for 20 min to retrieve the antigen. The slides were blocked with blocking buffer (5% bovine serum albumin, FA016-100G, Genview) for 30 min at 37°C after treatment with 3% hydrogen peroxide for 40 min. The IRF9 primary antibody (Proteintech, 14167-1-AP, at 1:500 dilution) was added to the slides for incubation overnight at 4°C after removing the blocking solution. The peroxidase-conjugated secondary antibody (Kit-9902, MXB Biotechnologies) was incubated for 30 min at 37°C. A DAB kit (DAB-0031, MXB Biotechnologies) was used to develop the color, and the sections were counterstained with hematoxylin. The slices were mounted with neutral resins (10004160, Sinopharm) after dehydration and observed under the microscope. The intensity of medial IRF9 staining was quantified relative to the area of the medial layer to evaluate the relative expression level of IRF9.

### Western Blot Analysis and Antibodies Information

Western blot (WB) analyses were performed as previously described (Jiang et al., 2016; Jiang et al., 2017a; Jiang et al., 2017b; Guo et al., 2019). PASMCs infected with the indicated lentivirus were starved for 12 h before treating with a specific stimulus, such as hypoxia or pharmacological intervention. Total protein was extracted from PASMCs and HPASMCs by RIPA lysis buffer [900 μL RIPA, 20 μL PMSF, 10 μL protease and phosphatase inhibitor cocktail (Thermo Fisher, 78440), 10 μL EDTA solution (AM9260G, Thermo Fisher Scientific), 50 μL NaF, 10 μL Na_3_VO_4_]. The Pierce™ BCA Protein Assay Kit (23225, Thermo Fisher Scientific) was used to determine the protein concentration. Fifteen micrograms of denatured protein were loaded and separated by SDS-PAGE, and then transferred to a polyvinylidene fluoride (PVDF) membrane (IPVH00010, Millipore). The membrane was incubated with primary antibodies against IRF9 (14167-1-AP; 1:1,000; rabbit; Proteintech), Flag (F1804; 1:1,000; mouse; Sigma), PCNA (GTX100539; 1:1,000; rabbit; Genetex), p-Histone H3 (sc-8656-R; 1:200; rabbit; Santa Cruz), β-Actin (#8457S; 1:1,000; rabbit; CST), p-p38 (#4511; 1:1,000; rabbit; CST), p38 (#8690; 1:1,000; rabbit; CST), P-ERK1/2 (#4370; 1:1,000; rabbit; CST), ERK1/2 (#4695; 1:1,000; rabbit; CST), DJ-1 (#11315; 1:1,000; rabbit; CST), GPX4 (ab125066; 1:1,000; rabbit; Abcam), PHB1 (#2426; 1:1,000; rabbit; CST), P-AKT^Thr308^ (#13038; 1:1,000; rabbit; CST), P-AKT^Thr3473^ (#4060; 1:1,000; rabbit; CST), AKT (#4685; 1:1,000; rabbit; CST), P-GSK3β (#12456; 1:1,000; rabbit, CST), GSK3β (#5558; 1:1,000; rabbit; CST) overnight at 4°C after blocked with 5% non-fat milk for 90 min at room temperature. Next day, the membrane was incubated with the peroxidase-conjugated secondary antibody (1:10000 dilution, 111-035-003, Jackson ImmunoResearch Laboratories) for 1 h at room temperature. The ChemiDocTM Touch Imaging System (Bio-Rad) was used to detect the protein signals and then analyzed by Image lab software (version 5.2.1, Bio-Rad).

### EdU Incorporation Assay

A Cell-Light™ Edu Apollo567 *In Vitro* kit (C10310-1, RiboBio) was used to perform the EdU incorporation assay. After overexpressing and knocking down of IRF9, RPASMCs and HPASMCs were plated in 12-well plates at 3 × 10^4^ cells per well and placed in cell incubators with indicated oxygen concentration (1% for hypoxia and 20% for normoxia) for 48 h. After incubating with 50 μM EdU medium (300 μL per well) for 2 h, the cells were fixed with 4% paraformaldehyde for 30 min and incubated with 2 mg/ml glycine to neutralize paraformaldehyde. After washing with PBS containing 0.5% Triton X-100 for 10 min, cells were incubated with 1× Apollo staining solution for 30 min. Finally, washing the cells with 0.5% Triton X-100 PBS solution again, and cells were incubated with 1× Hoechst 33342 for 30 min at room temperature. Fluorescence images of cells were collected under fluorescence microscope.

### CCK-8 Assay

A cell counting kit-8 (CCK-8) assay (CK04, Dojindo, Kumamoto, Japan) was used to assess the proliferation capacity of RPASMCs and HPASMCs. RPASMCs and HPASMCs transfected with indicated lentivirus were treated with hypoxia or normoxia (1% or 20% O_2_ concentration) for 48 h and then seeded in 96-well plates at a concentration of 8  ×  10^3^ cells per well. After washing with sterile PBS, cells were incubated with WST-8 [2-(2-methoxy-4-nitrophenyl)-3-(4-nitrophenyl)-5-(2,4-disulfophenyl)-2H-tetrazolium, monosodium salt] for 2 h. Then, the optical density value of absorbance at 450 nm was measured.

### Real-Time PCR

Real-time PCR was performed as previously reported (Jiang et al., 2014d; Li et al., 2018). Briefly, total mRNA was isolated by using TRI Reagent^®^ Solution (AM9738; ThermoFisher Scientific). Then, the mRNA was reversely transcribed into cDNA by using a transcriptor first strand cDNA synthesis kit (4896866001; Roche). The relative mRNA levels of target genes were detected by CFX connect™ real-time PCR detection system (Bio-Rad) using iQ™SYBR^®^ green supermix (1708884; Bio-Rad) and quantified relative to 18S. Primers used in this study were IRF9 forward primer 5′-TGT​AAG​CCA​CTC​AGA​CAG​CG-3′ and IRF9 reverse primer 5′-TCC​TCT​TGA​ACG​GTG​GCT​TC-3′, DJ-1 forward primer 5′-AGG​TTA​CAG​GGA​TAG​CCA​AAC​A-3′ and DJ-1 reverse primer 5′-CAG​GCT​CTC​AGT​GTC​AAG​CA-3′, GPX4 forward primer 5′-GAA​CCT​GGA​CGC​CAA​AGT​CCT​A-3′ and GPX4 reverse primer 5′-TTG​CTG​GTC​TGG​GGA​AGG​TC-3′, PHB1 forward primer 5′-CAG​AGC​GAG​CAG​CAA​CAT​TC-3′ and PHB1 reverse primer 5′-TCT​GGC​TCT​CTC​TGC​TTC​CT-3′, 18S forward primer 5′-CTC​AAC​ACG​GGA​AAC​CTC​AC-3′ and 18S reverse primer 5′-CGC​TCC​ACC​AAC​TAA​GAA​CG-3′.

### Luciferase Reporter Assay

HEK293T cells (3 × 10^4^) were seeded in 48-well plates, and when the confluence reached 35%, empty vector or IRF9 overexpression vectors (0.15 μg per well), as well as IRF9 shRNA vectors (0.3 μg per well), were transiently transfected in combination with PHB1 promoter-PGL3 (0.05 μg per well) and TK (0.02 μg per well). After 48 h, cells were lysed, and firefly and Renilla luciferase activities were measured using a dual-luciferase reporter assay kit (E1910, Promega). We calculated relative luciferase activity as the ratio of firefly (PHB1 promoter-PGL3) to Renilla (TK) activity. The experiments were performed in sextuplicate wells.

### Co-Immunoprecipitation Assays

Protein interactions between IRF9 and AKT were confirmed via co-immunoprecipitation (Co-IP), and western blotting was conducted with the indicated antibodies after Co-IP. Co-IP was performed in human pulmonary artery smooth muscle cells (HPASMCs) infected with Flag/IRF9-Flag lentivirus or untreated in three 10 cm dishes. Cells were lysed in 1 ml prelysis buffer (20 mM Tris-HCl, pH 7.4, 150 mM NaCl, 1 mM EDTA, 1% Triton X-100) with proteinase inhibitors (HY-K0010, MCE) per sample, and then sonicated (30% power output, 2 s, off 2 s, 15 times). The cell lysate was centrifuged at 12000 rpm at 4°C, for 15 min, and the supernatant was collected. Supernatant (100 μL) was reserved as input. For each immunoprecipitation, 850 μL supernatant was incubated with 1 μg of the indicated antibody and rotated at 4°C overnight. The next day, 35 μL magnetic beads (B23202, Biomake) were added to each sample and further incubated for 3 h in 4°C. Magnetic beads were collected after centrifugation, and the pellets was washing with washing buffer (0.5 M NaCl in prelysis buffer) 4 times. The coprecipitation product was eluted with 80 μL of 1× SDS loading buffer and denatured in 95°C for 15 min.

### Mitochondrial Function Evaluation

JC-1 mitochondrial membrane potential assay kit (C2003S, Beyotime) was used to detect the mitochondrial membrane potential. The HPASMCs were transfected with indicated lentivirus and treated with indicated oxygen concentration stimulations as mentioned above were seeded into 24-wells plates. The cells were washed with PBS for once, and incubated with 500 μL staining working buffer per well for 20 min, and the covering liquid was changed to DMEM/F12 after washing cells with staining buffer solution for twice, and the cell-fluorescence were observed by fluorescence microscope.

The mitochondrial permeability transition pore (mPTP) assay kit (C2009S, Beyotime) was used to detect opening of mPTP. After processing accordingly, HPASMCs were seed into 24-wells plates. The cells were incubated with 500 μL fluorescence quenching solution per well for 30 min, and then change the incubated liquid to pre-warmed DMEM/F12 for 30 min under light avoiding condition. The cell-fluorescence were observed under fluorescence microscope after washing with PBS for twice.

### Statistical Analysis

The data were analyzed by SPSS (version 22.0.0, SPSS Inc., Chicago, IL, United States) or Prism (version 8.3.0, GraphPad software, LLC) and presented as the mean ± SD, A Two-tailed independent sample *t*-test was used to compare the difference between two groups. One-way or two-way ANOVA was used to compare the differences between three or more groups. *p* < 0.05 was considered statistically significant.

## Results

### The Expression Level of IRF9 Is Upregulated in the Thickened Pulmonary Arterioles of PAH Rat and Mouse Models

**FIGURE 1:**
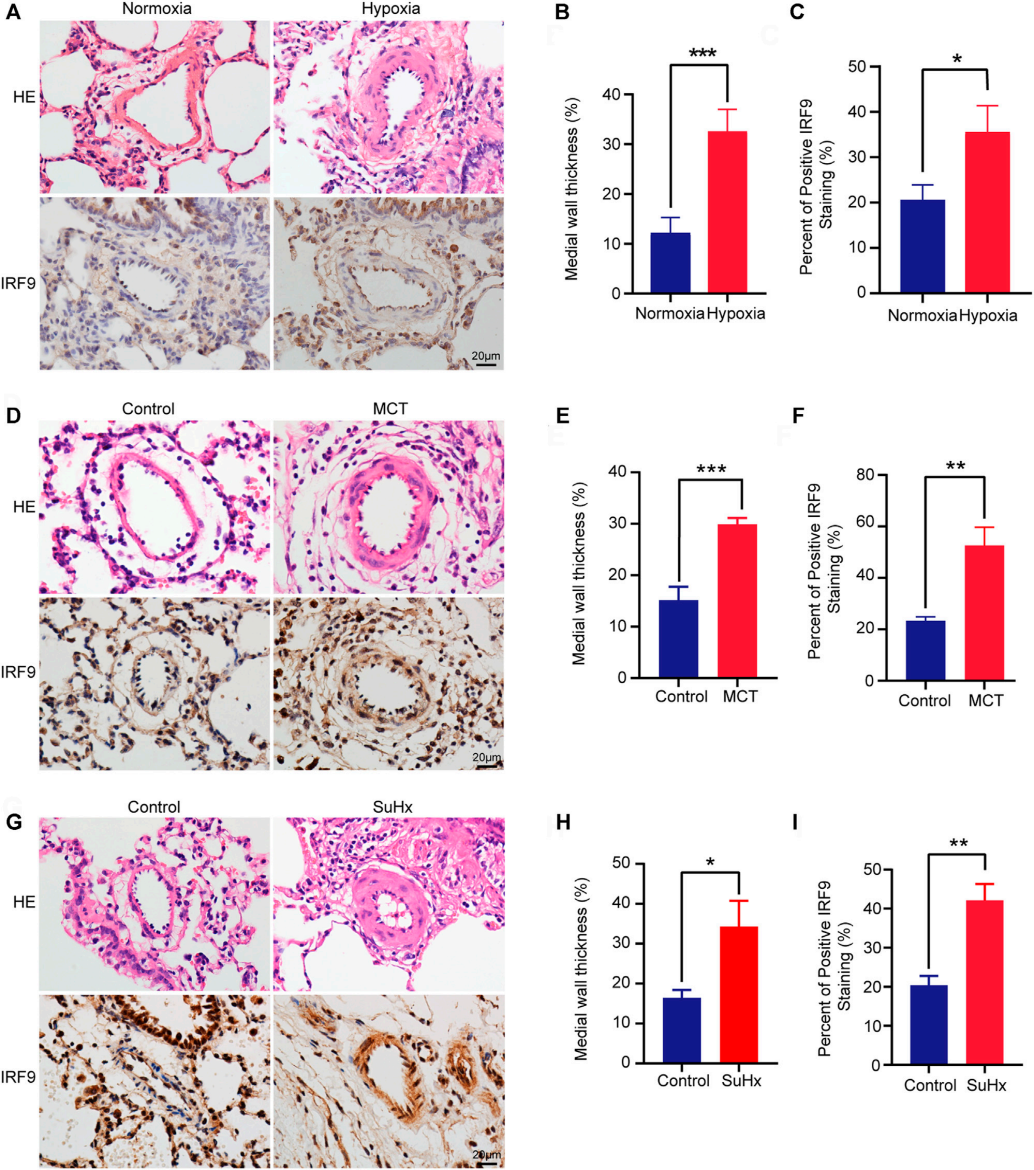
IRF9 expression is upregulated in the thickened medial wall of the pulmonary artery in chronic hypoxia, MCT and SuHx rat PAH models. **(A)** The representative pulmonary arteriole with Haematoxylin and eosin (HE) staining (the upper panel) and immunohistochemical staining of IRF9 (the lower panel) in the lung sections of control group and chronic hypoxia-induced rat PAH model. Scale bar = 20 μm. **(B)** The statistics of medial wall thicknesses [inner diameter/(inner diameter + outside diameter)] of pulmonary arteriole in **(A)** (*n* = 3 rats per group). **(C)** According to the results of immunohistochemical staining, the intensity of medial IRF9 staining was quantified relative to the area of the medial layer (*n* = 3 rats per group). **(D**–**F)** The representative images of pulmonary arteriole with HE and IRF9 staining in MCT-induced rat PAH model **(D)**, quantification of medial wall thicknesses **(E)** and IRF9 expression level **(F)** (Scale bar = 20 μm, *n* = 3 rats per group). **(G**–**H)** In control and SuHx-induced rat PAH model, HE staining was used to show the remodeling of the pulmonary arterioles **(G)**, and the degree of thickening is quantified in **(H)**; the expression of IRF9 in the pulmonary arterioles was detected by immunohistochemical staining **(G)** and was quantified **(I)** Scale bar = 20 μm. (*n* = 3 rats per group). Values are means ± SD; ***p < 0.001, **p < 0.01, *p < 0.05.

To investigate the involvement of IRF9 in PAH, we generated a variety of PAH models of chronic hypoxia (CH)-induced rats or mice, and monocrotaline (MCT)-, sugen5416 combined with chronic hypoxia (SuHx)-induced rats. The results showed that right ventricle systolic pressure (RVSP), Fulton’s mass index (RV/LV + S), right ventricular wall thickness at end-diastole (RVWd), right ventricular wall thickness at end-systole (RVWs), interventricular septal thickness at diastole (IVSd), and interventricular septal thickness at systole (IVSs) were increased in hypoxia-treated rats compared with normoxia-treated rats (Supplementary Figures S1A–C), while right ventricular inside diameter at end-diastole (RVIDd) and right ventricular inside diameter at end-systole (RVIDs) showed no significant difference between groups (Supplementary Figure S1C). Furthermore, HE staining revealed thickening of the medial layer of pulmonary arterioles in rats after exposure to hypoxia for 4 weeks (Figures 1A,B). These results indicated that severe PAH and RHF were induced by hypoxia in rats. Furthermore, a higher IRF9 expression level was observed in the pulmonary arteriole medial wall of hypoxia-treated rats than in normoxia-treated rats (Figures 1A,C). Moreover, in MCT- and SuHx-induced PAH rat models, the results of HE staining showed that obvious pulmonary arteriole remodeling was observed (Figures 1D,E,G,H). Consistent with hypoxia-induced PAH, IRF9 was increased in pulmonary arteriole of rats treated with MCT or SuHx compared with their counterparts (Figures 1D,F,G,I).

Consistently, in a mouse PAH model induced by chronic hypoxia treatment for 4 weeks, RVSP, Fulton’s mass index, RVWs, and RVIDs were significantly increased compared with those of the normoxia mice, while comparable diameters of RVWd, RVIDd, IVSd and IVSs were observed between hypoxia- and normoxia-treated mice (Supplementary Figures S1D–F). HE staining revealed that the medial wall of pulmonary arterioles in hypoxia-treated mice was remarkably thickened (Supplementary Figures S1G, H). More importantly, by using immunohistochemical (IHC) staining, we found that IRF9 expression was increased in a time-dependent manner in the thickened pulmonary arteriole medial wall of mice exposed to hypoxia for 1, 2, 3 and 4 weeks compared with the mice in the normoxia group (Supplementary Figures S1I, J).

In addition, compared with normoxia group, the protein level of IRF9 was dramatically increased in HPASMCs treated with 1% oxygen for 6 and 24 h **(** Supplementary Figures S2A, B **)**.

Hence, our results demonstrated that the expression level of IRF9 was elevated in the thickened medial wall of pulmonary arterioles in multiple stimulations-induced *in vivo* and *in vitro* PAH models, which indicated that IRF9 may be involved in the process of pulmonary arteriole medial wall hyperplasia and PASMC proliferation during PAH formation.

### Overexpression of IRF9 Promotes the Proliferation of RPASMCs

**FIGURE 2:**
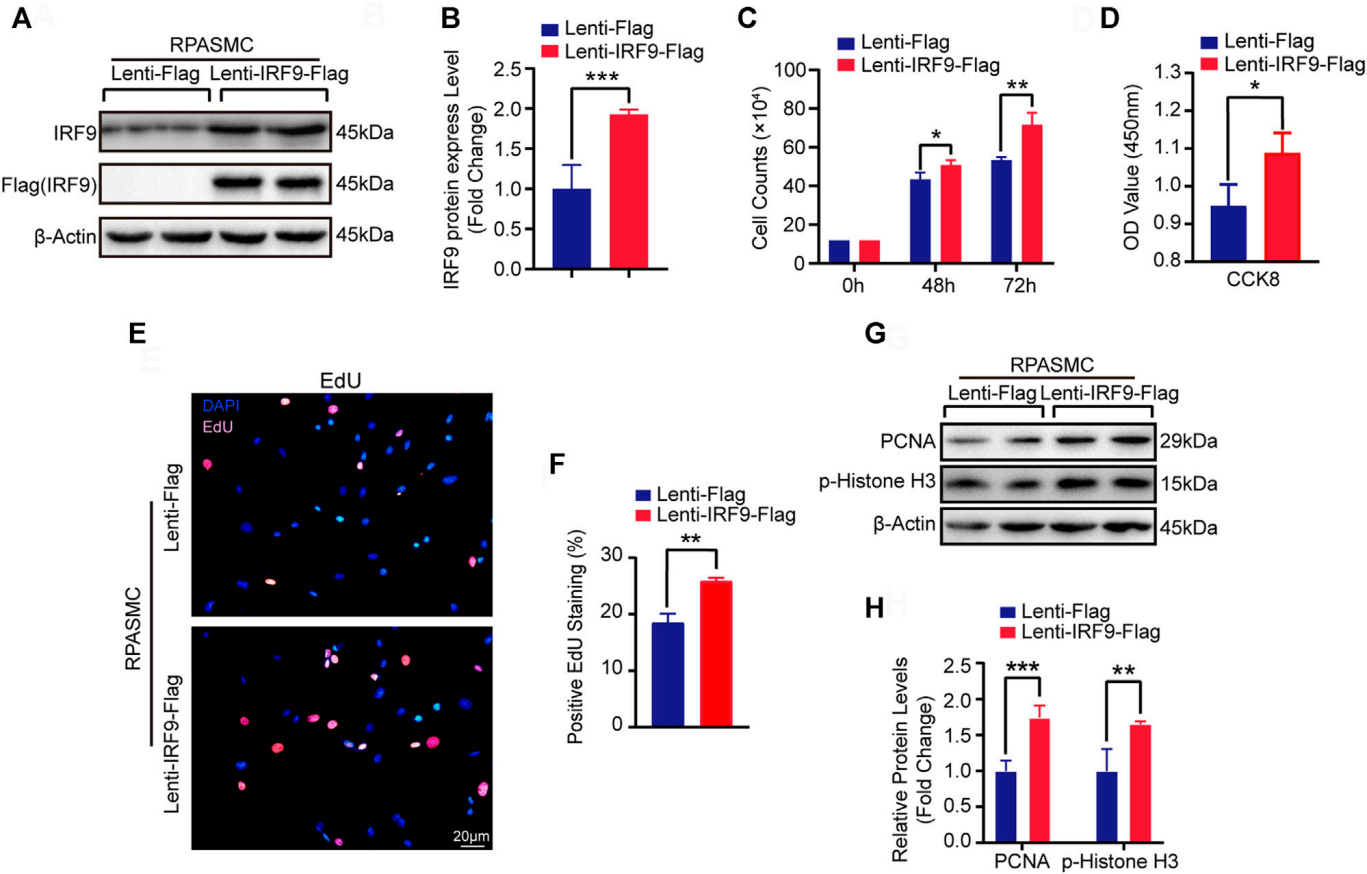
IRF9 overexpression increased the proliferation of RPASMCs. **(A)** After overexpressing IRF9 (Lenti-IRF9-Flag) or not (Lenti-Flag) in RPASMCs, the expression level of IRF9 and the exogenous label protein Flag was determined using western blot (*n* = 3 independent experiments). **(B)** The expression levels of IRF9 were normalized to β-Actin and quantified (*n* = 3 independent experiments). **(C)** Cell numbers were counted at indicated time point after IRF9-overexpressing or not in RPASMCs under normoxia condition (*n* = 3 independent experiments). **(D)** CCK-8 assay was performed and the absorbance (OD value) at 450 nm was measured to shows the proliferation activity of Lenti-Flag and Lenti-IRF9-Flag RPASMCs under normoxia condition (*n* = 3 independent experiments). **(E)** EdU incorporation assay shows the ratio of cells incorporated EdU in Lenti-Flag and Lenti-IRF9-Flag RPASMCs under normoxia condition, in which nuclei were stained with DAPI (blue) and EdU merged with DAPI appears pink. Scale bar = 20 μm. **(F)** The positive EdU staining rates were quantified (*n* = 3 independent experiments). **(G)** The protein levels of proliferation biomarkers PCNA and p-Histone H3 were determined using western blot after overexpressing IRF9 or not in RPASMCs under normoxia condition. **(H)** The expression levels of PCNA and p-Histone H3 were quantified and normalized to β-Actin (*n* = 3 independent experiments). Values are means ± SD; ***p < 0.001, **p < 0.01, *p < 0.05.

Since the expression level of IRF9 was increased in *in vivo* and *in vitro* models of PAH whose major pathological feature is PASMC proliferation, we were curious about whether overexpression of IRF9 induced the proliferation of PASMCs. Thus, to further investigate the function of IRF9 in regulating the proliferation of PASMCs, we constructed IRF9 overexpression plasmids that were transfected into primary RPASMCs via lentiviruses (Figures 2A,B). Compared with RPASMCs infected with Lenti-Flag (control), IRF9 overexpression obviously accelerated RPASMC growth after 48 and 72 h of culture under normal oxygen conditions (Figure 2C). In addition, the results of EdU incorporation assays and cell counting kit-8 (CCK-8) proliferation assays demonstrated that IRF9 overexpression obviously accelerated the proliferation of RPASMCs (Figures 2D–F). Furthermore, western blot analysis showed that the expression levels of proliferating cell nuclear antigen (PCNA) and phosphorylated histone H3 (p-Histone H3) which are the biomarkers of cell proliferation, were elevated in RPASMCs overexpressing IRF9 (Figures 2G,H). Together, these results suggested that increased IRF9 expression significantly promoted the RPASMC proliferation.

### Knockdown of IRF9 Inhibits the Proliferation of RPASMCs

**FIGURE 3:**
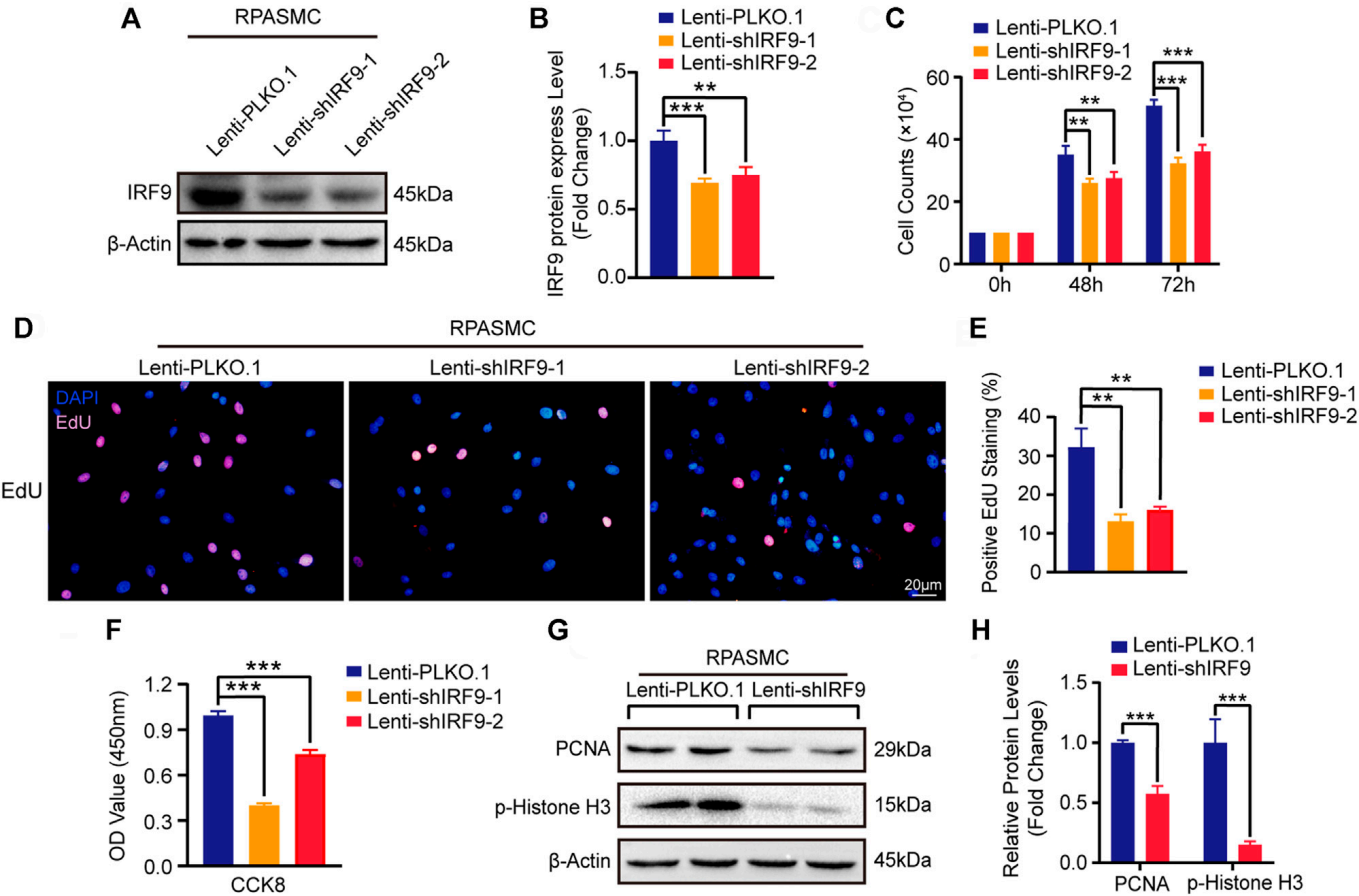
IRF9 knockdown decreases the proliferation of RPASMCs. **(A)** The expression level of IRF9 was verified by western blot after knockdown IRF9 by infected indicated lentiviruses (Lenti-shIRF9-1, Lenti-shIRF9-2) or not (Lenti-PLKO.1) in RPASMCs under normoxia condition. **(B)** The protein level of IRF9 were normalized to β-Actin and quantified (*n* = 3 independent experiments). **(C)** Cell counting assay shows the count of RPASMCs with Lenti-PLKO.1 or Lenti-shIRF9-1/2 at time point culturing for 0, 48 and 72 h under normoxia condition (*n* = 3 independent experiments). **(D)** EdU incorporation assay shows the ratio of RPASMCs incorporated with EdU after infected with Lenti-shIRF9-1/2 or Lenti-PLKO.1 under normoxia condition, in which nuclei are stained with DAPI (blue) and incorporated EdU merged with DAPI are shown in pink. Scale bar, 20 μm. **(E)** The positive EdU staining rates were measured (*n* = 3 independent experiments). **(F)** CCK-8 assay shows the decrease of absorbance at 450 nm which indicate the attenuation of proliferation capacity in Lenti-shIRF9-1/2 RPASMCs comparing to Lenti-PLKO.1 under normoxia condition (*n* = 3 independent experiments). **(G)** The protein levels of proliferation biomarkers PCNA and p-Histone H3 were determined in IRF9-knockdown RPASMCs in normoxia condition. **(H)** The expression levels of PCNA and p-Histone H3 were normalized to β-Actin and quantified (*n* = 3 independent experiments). Values are means ± SD; ***p < 0.001, **p < 0.01.

Next, we generated two different short hairpin RNA plasmids to knockdown IRF9 (shIRF9-1 and shIRF9-2) and tested the expression level of IRF9 in primary RPASMCs after infection with lentiviruses (Figures 3A,B). Compared with the control group (Lenti-PLKO.1), both Lenti-shIRF9-1 and Lenti-shIRF9-2 significantly suppressed the growth of RPASMCs, as evidenced by the growth curve (Figure 3C). The results of EdU incorporation assay and CCK-8 assay also supported the results that knockdown of IRF9 obviously restrained the proliferation of RPASMCs (Figures 3D–F). The expression levels of the proliferation biomarkers PCNA and p-Histone H3 were largely inhibited by IRF9 knockdown (Figures 3G,H). These results indicated that inhibition of IRF9 expression could be a novel strategy to retard RPASMC proliferation and alleviate PAH.

### IRF9 Facilitates the Proliferation of Human PASMCs

**FIGURE 4:**
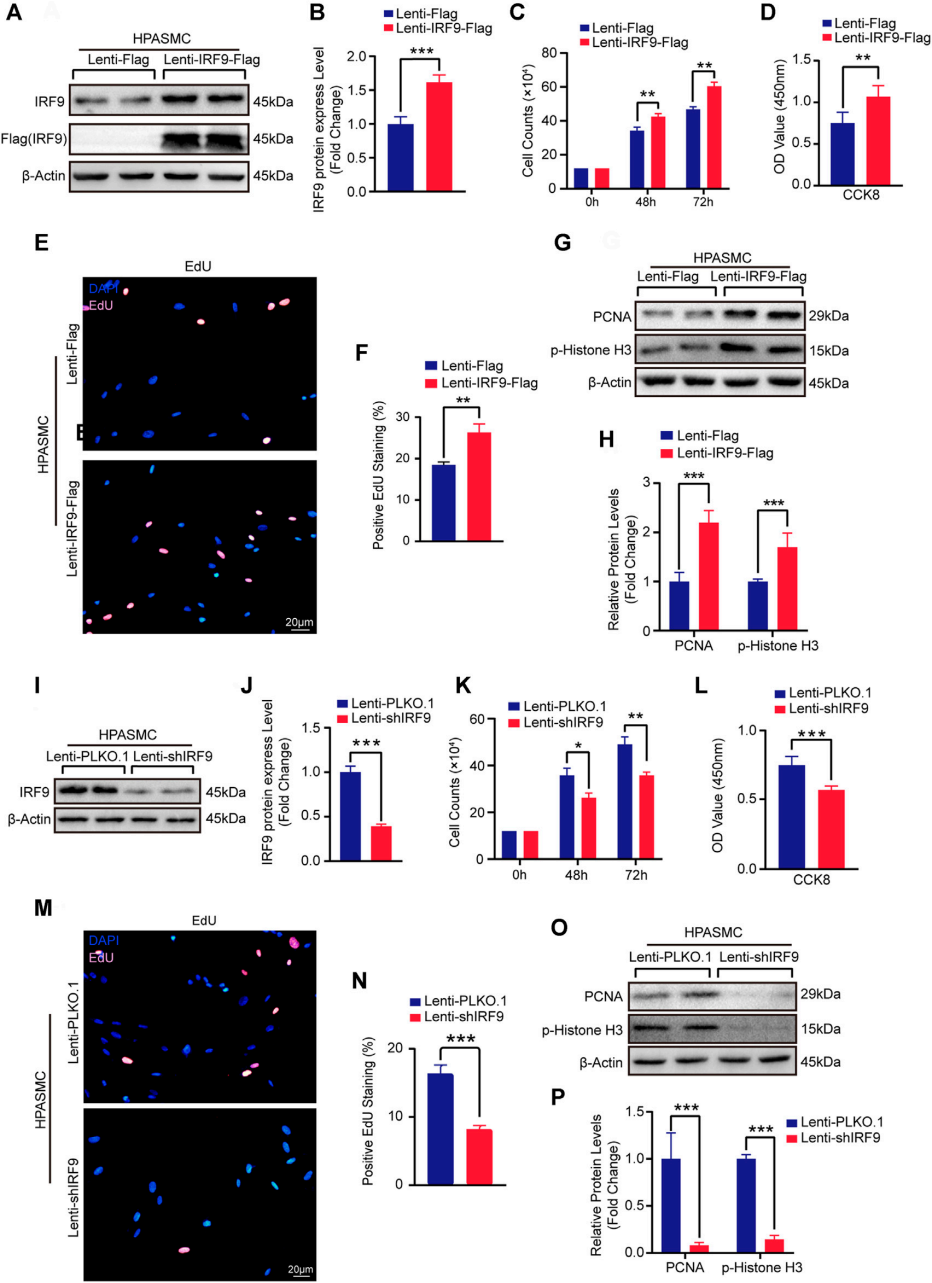
Upregulation of IRF9 accelerate and downregulation attenuate the proliferation of HPASMCs. **(A,B,I and J)** The protein level of IRF9 in HPASMCs with IRF9 overexpression **(A)** or knockdown **(I)** were detected by using western blot (*n* = 3 independent experiments); the IRF9 protein level was quantified **(B,J)**. **(C,K)** The counts of cell number after HPAMSCs treated with Lenti-IRF9-Flag **(C)** or Lenti-shIRF9 **(K)** for indicated time under normoxia condition; Lenti-Flag and Lenti-PLKO.1 serve as control group (*n* = 3 independent experiments). **(D,L)** CCK-8 assay shows the absorbances at 450 nm of Lenti-Flag/Lenti-PLKO.1 and Lenti-IRF9-Flag/Lenti-shIRF9 HPASMCs under normoxia condition (n = 3 independent experiments). **(E,M)** EdU incorporation assay of HPASMCs infected with Lenti-Flag/Lenti-IRF9-Flag **(E)** or Lenti-PLKO.1/Lenti-shIRF9 (M) under normoxia condition, in which nuclei were stained with DAPI (blue) and EdU merged with DAPI appears pink. Scale bar, 20 μm. **(F,N)** The positive EdU staining rate was measured in HPASMCs after IRF9 overexpression **(F)** or knockdown (N) (*n* = 3 independent experiments). **(G,O)** Protein levels of PCNA and p-Histone H3 were determined by western blot after overexpressing (G) or knockdown **(O)** of IRF9 in HPASMCs under normoxia condition. **(H,P)** The expression levels were normalized to β-Actin and quantified (*n* = 3 independent experiments). β-Actin serves as loading control. Values are means ± SD; ***p < 0.001, **p < 0.01, *p < 0.05.

In order to further explore the possibility of IRF9 affecting the occurrence of human PAH, we overexpressed or knocked down IRF9 in HPASMCs (Figures 4A,B,I,J). The results showed that compared with Lenti-Flag, IRF9 overexpression remarkably promoted the proliferation of HPASMCs, as evidenced by increased cell numbers (Figure 4C and Supplementary Figure S2C **)**, cell viability (Figure 4D and Supplementary Figure S2D), EdU positive cells (Figures 4E,F and Supplementary Figure S2E), as well as higher protein levels of proliferation biomarkers, PCNA and p-Histone H3 (Figures 4G,H and Supplementary Figure S2F) under both normoxia and hypoxia conditions. In contrast, the results of cell counting, EdU incorporation assay, CCK8 assay and western blotting for PCNA and p-Histone H3 demonstrated that IRF9 knockdown suppressed the proliferation of HPASMCs treated with normoxia or hypoxia (Figures 4K–P and Supplementary Figures S3A–D). Therefore, our results suggested that IRF9 may contribute to human PAH by regulating PASMCs proliferation.

### IRF9 Interacts With AKT to Affect PASMC Proliferation

**FIGURE 5:**
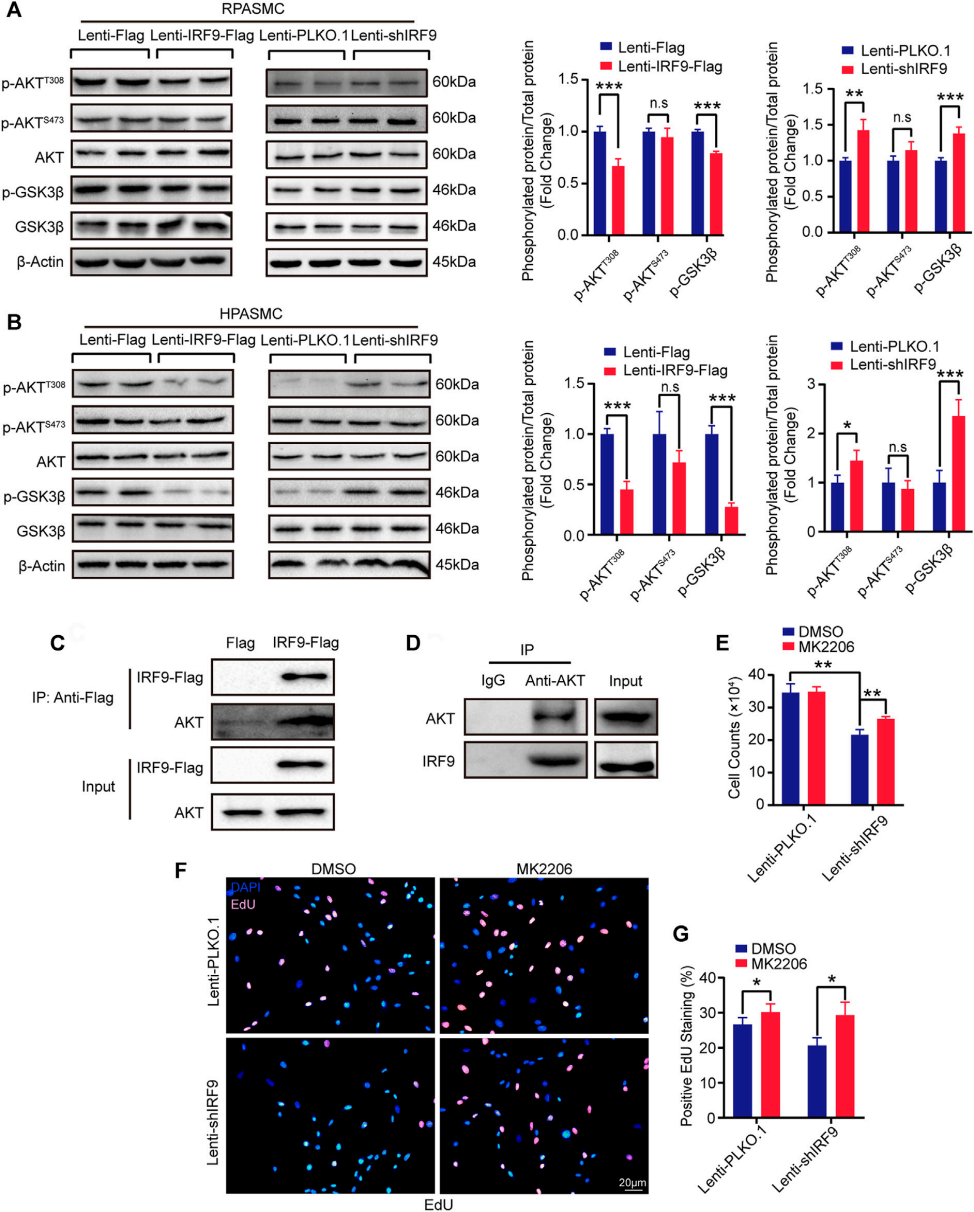
IRF9 regulates PASMC proliferation through the AKT/GSK3β pathway. **(A,B)** Representative western blots **(left panel)** and quantification results **(right panel)** of p-AKT^T308^, p-AKT^S473^, AKT, p-GSK3β and GSK3β in IRF9-overexpressed (Lenti-IRF9-Flag) or knocked down (Lenti-shIRF9) RPASMCs **(A)** and HPASMCs **(B)** under normoxia condition. The relative levels of phosphorylated proteins were normalized by their total protein respectively (*n* = 3 independent experiments). β-Actin serves as loading control. **(C,D)** Co-immunoprecipitation results show that exogeneous IRF9 interacts with endogenous AKT **(C)** and that endogenous AKT interacts with endogenous IRF9 **(D)** in HPASMCs. **(E–G)** The cell counts **(E)**, EdU incorporation staining **(F)** and quantification **(G)** of RPASMCs with IRF9 knockdown (Lenti-shIRF9) or not (Lenti-PLKO.1) treated with MK2206 (AKT phosphorylation inhibitor, 0.5 μM, 48 h) or DMSO; the nuclei were stained with DAPI (blue) and incorporated EdU merged with DAPI appears pink **(F)** (*n* = 3 independent experiments). Values are means ± SD; ***p < 0.001, **p < 0.01, *p < 0.05, n.s indicates non significance.

Given that many signaling pathways are involved in PAH, such as the mitogen-activated protein kinase (MAPK) and AKT signaling pathways (J. Dai et al., 2018; Y. Guo et al., 2020b), we wondered whether IRF9 could regulate these pathways to affect the proliferation of PASMCs. Thus, we first assessed the phosphorylation levels of ERK1/2 and p38 in RPASMCs, which are the key components of the MAPK signaling pathway. Our results showed that compared with the control group, neither IRF9 overexpression nor IRF9 knockdown affected the phosphorylation levels of either ERK1/2 or p38 in RPASMCs (Supplementary Figure S4A). However, IRF9 overexpression significantly inhibited the phosphorylation of AKT^Thr308^ but not AKT^Ser473^, and its downstream GSK3β was also hypophosphorylated compared with cells infected with Lenti-Flag, and IRF9 knockdown accelerated the phosphorylation of AKT^Thr308^ and GSK3β in both RPASMCs and HPASMCs under normoxia condition (Figures 5A,B). Consistent with normoxia treatment, IRF9 inhibited the phosphorylation of AKT^Thr308^ and GSK3β under hypoxia treatment in HPASMCs (Supplementary Figure S4B). These results indicated that IRF9 inhibited the AKT-GSK3β signaling pathway in PASMCs under both normoxia and hypoxia conditions.

Next, co-immunoprecipitation (co-IP) was performed to investigate whether IRF9 interacted directly with AKT to regulate the AKT-GSK3β signaling pathway. Our results demonstrated that IRF9 interacted with AKT under both exogenous and endogenous conditions, and *vice versa* (Figures 5C,D). Furthermore, MK2206, an inhibitor of the AKT signaling pathway, obviously increased the cell number and EdU positive cells which were inhibited by IRF9 knockdown (Figures 5E–G). Thus, these results suggested that IRF9 interacted with AKT and then inhibited the phosphorylation of AKT^Thr308^ and GSK3β to promote the proliferation of PASMCs.

### IRF9 Promotes the Proliferation of PASMCs by Downregulating PHB1 Expression

**FIGURE 6:**
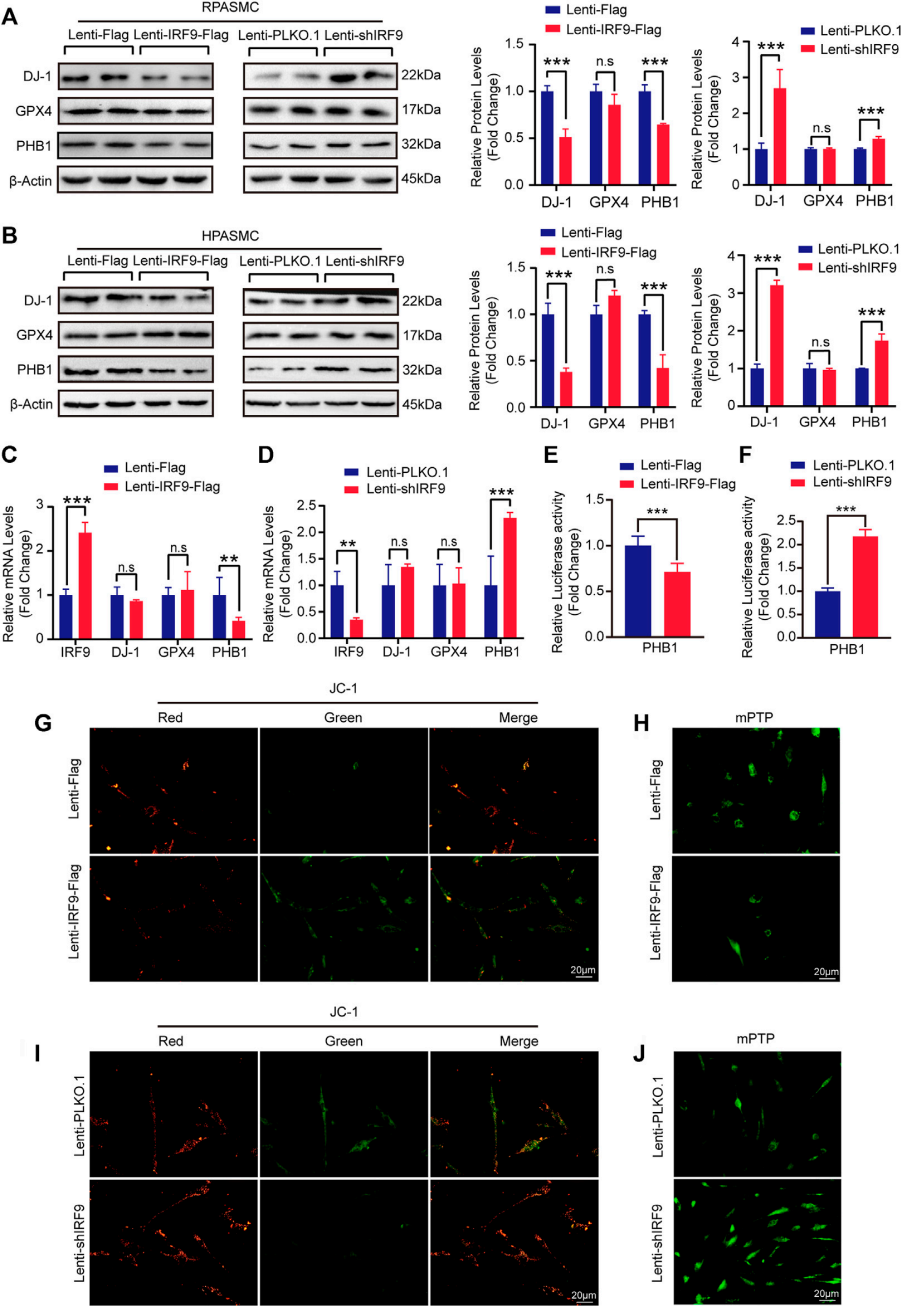
IRF9 affects the mitochondrial function through regulating PHB1 expression. **(A,B)** The protein levels of DJ-1, GPX4 and PHB1 in IRF9-overexpressing (Lenti-IRF9-Flag), IRF9-knockdown (Lenti-shIRF9) and neither (Lenti-Flag/Lenti-PLKO.1) RPASMCs **(A)** and HPASMCs **(B)** were assessed by western blotting **(left panels)** under normoxia condition. Proteins levels were quantified and normalized to β-Actin **(right panels)** (*n* = 3 independent experiments). **(C,D)** The mRNA levels of IRF9, DJ-1, GPX4 and PHB1 in HPASMCs with IRF9 overexpression **(C)** or knockdown **(D)** were detected by RT-PCR (*n* = 3 independent experiments). **(E,F)** The luciferase activity controlled by PHB1 promoter was evaluated when IRF9 overexpression **(E)** or knockdown **(F)** (*n* = 6 independent experiments). **(G,I)** The representative images of mitochondrial membrane potential detected by using JC-1 kit in HPASMCs with IRF9 overexpression **(G)** or knockdown **(I)** under normoxia condition. Scale bar, 20 μm (*n* = 3 independent experiments). **(H, J)** The openness of mitochondrial permeability transition pore (mPTP) is detected by the mPTP kit in HPASMCs with IRF9 overexpression **(H)** or knockdown **(J)** under normoxia condition, Scale bar, 20 μm (*n* = 3 independent experiments). Values are means ± SD; ***p < 0.001, **p < 0.01, n.s indicates non significance.

Given that AKT signaling pathway participates in regulating mitochondrial function during PAH development (Robey and Hay, 2009), we were curious about whether IRF9 plays a role in regulating mitochondrial function. Mitochondrial dysfunction contributes to the metabolic transformation of PASMCs from a quiescent state to a hyperproliferative state during PAH (Leopold and Maron, 2016; Vander Heiden et al., 2009; Marshall et al., 2018). Glutathione peroxidase 4 (GPX4) and DJ-1 are involved in the maintenance of mitochondrial reactive oxygen species (ROS) levels, since excessive ROS can damage mitochondria (Gao et al., 2019; J. Zeng et al., 2019). We found that compared with the control, the expression level of DJ-1 was inhibited by IRF9 overexpression but promoted by IRF9 knockdown in both RPASMCs and HPASMCs, and comparable levels of GPX4 were detected in both RPASMCs and HPASMCs with or without IRF9 overexpression or knockdown under normoxia condition (Figures 6A,B). Similar results were observed in HPASMCs treated with hypoxia **(** Supplementary Figure S5A **)**. As IRF9 is a transcript factor (Jiang et al., 2014c), we further detected the mRNA levels of DJ-1 and GPX4. The results showed that neither IRF9 overexpression nor knockdown affected the mRNA levels of GPX4 and DJ-1 (Figures 6C,D), which indicated that DJ-1 and GPX4 were not the direct targets regulated by IRF9.

Prohibitin 1 (PHB1) was reported to not only function as a chaperone protein that stabilizes mitochondrial respiratory enzymes and maintains mitochondrial integrity but also have anti-proliferative activity (S. D. Guo SD. et al., 2020; Cao et al., 2020). Thus, we assessed the expression level of PHB1 in RPASMCs and HPASMCs with overexpression or knockdown of IRF9. Our results showed that the protein level of PHB1 was significantly inhibited by IRF9 overexpression but elevated by IRF9 knockdown in both RPASMCs and HPASMCs under normoxia condition (Figures 6A,B), as well as in HPASMCs treated with hypoxia **(** Supplementary Figure S5A **)**. Unlike DJ-1 and GPX4, the mRNA level of PHB1 was obviously suppressed by overexpression of IRF9, but enhanced by IRF9 knockdown (Figures 6C,D). Moreover, the results of luciferase reporter gene harboring the promoter region of PHB1 demonstrated that IRF9 can inhibit the expression of PHB1 at transcriptional level (Figures 6E,F). Therefore, these results indicated that IRF9 can directly suppress the expression of PHB1.

Given that both AKT signaling pathway and PHB1 were regulators of mitochondrial function, we further evaluated the mitochondrial function by using JC-1 mitochondrial membrane potential assay kit and mitochondrial permeability transition pore (mPTP) assay kit in IRF9 knockdown and overexpressed HPASMCs. The normal cells have high level of mitochondrial membrane potential (MMP) (red fluorescence) and extremely low degree of mPTP opening (abundant green fluorescence). In our results, IRF9 overexpression results in a depolarization and decrease of MMP (green fluorescence) and abnormal opening (quench of green fluorescence) which indicated the damage of mitochondrial function (Figures 6G,H and Supplementary Figures S5B, C). On the contrary, IRF9 knockdown led to elevation of MMP and decrease of mPTP opening indicating a protecting affection to mitochondrial function under both normoxia and hypoxia condition (Figures 6I,J and Supplementary Figures S2D, E). Overall, our results demonstrated that IRF9 contributes to mitochondrial dysfunction by inhibiting PHB1 expression to promote the proliferation of PASMCs.

## Discussion

Pulmonary vascular remodeling is a common pathophysiological process that occurs in PAH, and the abnormal proliferation of PASMCs upon stimulation by endogenous and exogenous insults is of prime importance in this process (Leopold and Maron, 2016). However, the mechanisms underlying these PASMC responses remain poorly understood. In the present study, we revealed that the IRF9 expression level was elevated in the pulmonary arteriole medial wall of mice and rats exposed to multiple PAH inducers. The proliferation of PASMCs was promoted by overexpression of IRF9 and inhibited by knockdown of IRF9. Furthermore, we found that IRF9 not only directly suppressed PHB1 expression but also interacted with AKT to restrain the phosphorylation of AKT^Thr308^ and GSK3β to affect PASMC proliferation. Based on the results of our present study, we proposed an “IRF9-PHB1-AKT axis” that can be considered a therapeutic target for the prevention of pulmonary vascular remodeling and PAH.

In the search for ways to effectively prevent the progression of PAH, tremendous efforts have been made in recent decades to elucidate the pathogenesis and mechanism of pulmonary vascular remodeling. Remarkably, cases of interferon-induced PAH suggest a causal link between interferon (IFN) exposure and PAH (Al-Zahrani et al., 2003; Jochmann et al., 2005; Ledinek et al., 2009; Dhillon et al., 2010; Savale et al., 2014); subsequently, basic experiments validated the interaction between IFN and PH (Badiger et al., 2012; George et al., 2014). Furthermore, by knocking out the type I interferon receptor [interferon alpha receptor 1 (IFNAR1)^(−/−)^] in mice, *Peter et al.* demonstrated that blocking the type I IFN signaling pathway attenuates the progression of vascular remodeling and right heart failure (George et al., 2014). As a member of the IFN regulator factor (IRF) family, IRF9 is a transcription factor that mediates the type I IFN gene induction by activating distinct types of innate pattern-recognition receptors (Negishi et al., 2018) and usually plays pivotal roles in the immune response and inflammation (Jefferies, 2019). However, whether IRF9 regulates pulmonary vascular remodeling has never been investigated. In the current study, we explicitly determined that IRF9 expression levels were elevated in chronic hypoxia-, MCT- and SuHx-induced PAH rat models and hypoxia-induced mouse model. Although the related contribution of cells with different origins (for example, endothelial cells and adventitial fibroblasts) to pulmonary vascular remodeling remains to be determined (Schermuly et al., 2011), our results strongly suggested that IRF9 is predominantly involved in regulating arteriole medial smooth muscle layer thickening, and moreover, subsequent functional experiments by overexpressing and knocking down IRF9 in RPASMCs and HPASMCs substantiated that IRF9 is particularly important in PASMC proliferation. Similarly, our previous results also demonstrated that IRF9 facilitated the proliferation of carotid artery SMCs and injury-induced neointima formation, which further validated the results in this study (S. M. Zhang et al., 2014a). These results suggested that IRF9 is a hyperproliferative factor and that targeting IRF9 is a potential treatment for SMC proliferative diseases, such as PAH and coronary restenosis.

Similar to cancer cells, PASMCs in PAH exhibit a metabolic reprogramming referred to as the “Warburg effect”, in which the metabolic shift from glucose oxidation toward uncoupled aerobic glycolysis, leads to a hyperproliferative state contributing to the progression of pulmonary vascular remodeling (Peng et al., 2016). As the center of energy metabolism, mitochondria are directly involved in the metabolic shift toward aerobic glycolysis under external stimuli such as hypoxia (Ryan et al., 2015). In addition, mitochondria are also oxygen sensors in lung circulation, altering the cytosolic redox state through the production of reactive oxygen species (ion channels and kinases), which regulate the effectors that mediate hypoxic pulmonary vasoconstriction (Weir et al., 2005). Hence, acquired mitochondrial abnormalities in PASMCs disrupt oxygen sensing and create a pseudo-hypoxic environment, inducing PASMC metabolic reprogramming and pulmonary vasoconstriction and vascular remodeling. As an oxidative stress sensor and as an antioxidant, DJ-1 exhibits the properties of molecular chaperone, protease, and transcriptional regulator that protects mitochondrial function from oxidative stress (Dolgacheva et al., 2019). Although we found that the protein level of DJ-1 was affected by IRF9 in PASMCs, neither IRF9 overexpression nor knockdown altered the mRNA level of DJ-1, which indicated that the protein stability of DJ-1 may indirectly regulated by IRF9.

In humans, PHB1, the N-terminus of which is necessary for mitochondrial localization, maintains the normal function of mitochondria and is implicated in the assembly and activity of the oxidative phosphorylation system (OXPHOS), mitochondrial biogenesis, and mitochondrial networks (Signorile et al., 2019). In the present study we found that IRF9 is the transcription factor regulating PHB1 expression in the process of PASMC proliferation. In cancer cells, depletion of PHB1 causes defects in proliferation (Cheng et al., 2020), but little is currently known about the regulation of PHB1 in pulmonary vascular remodeling and PAH. Here, we fill this knowledge gap by demonstrating that IRF9 negatively regulates PHB1 expression upon PASMC proliferation. Reduced PHB1 expression attenuates the protection of mitochondrial function and disturbs mitochondrial oxidative stress, the oxidative phosphorylation system and the glycolytic metabolic shift, leading to cancer-like hyperproliferation phenotype, ultimately resulting in pulmonary vascular remodeling and PAH.

AKT is considered to be the downstream target of PHB1 in regulating mitochondrial function (X. L. Zhang et al., 2019; Yang et al., 2005; Mishra et al., 2010). Studies on mitochondria regulating energy metabolism show that activation of AKT increases the total cellular ATP content by content by 2-3-fold (Hahn-Windgassen et al., 2005), and oxygen consumption is elevated in cells expressing activated AKT, but reduced in AKT-deficient cells (Nogueira et al., 2008). In order to serve as original carbon materials for macromolecular synthesis, glucose cannot be committed to carbon catabolism for ATP production. For proliferative cells, a high ATP/ADP ratio is disadvantageous (Vander Heiden et al., 2009), and AKT deficiency induces a reduction in ATP/ADP, which suggests a cellular glucose metabolism shift from aerobic respiration to glycolysis, so that glucose is allowed to provide carbon, nitrogen, and free energy to generate biomass (Vander Heiden et al., 2009). Because of this, AKT is also known as the “Warburg kinase” (Robey and Hay, 2009). In the present study, our results reveal that on the one hand, IRF9 directly regulates the expression of PHB1 and also directly interacts with AKT to inhibit the phosphorylation of AKT at the Thr^308^ site but not at the Ser^473^ site; on the other hand, IRF9 overexpression results in mitochondrial dysfunction by increasing opening of mPTP and mitochondrial permeability. Given the important regulatory role of PHB1 and AKT in mitochondrial function, our results indicated that IRF9 may regulate mitochondrial function and energy metabolism to promote the proliferation of PASMCs in PAH via the IRF9- PHB1-AKT axis.

**FIGURE 7:**
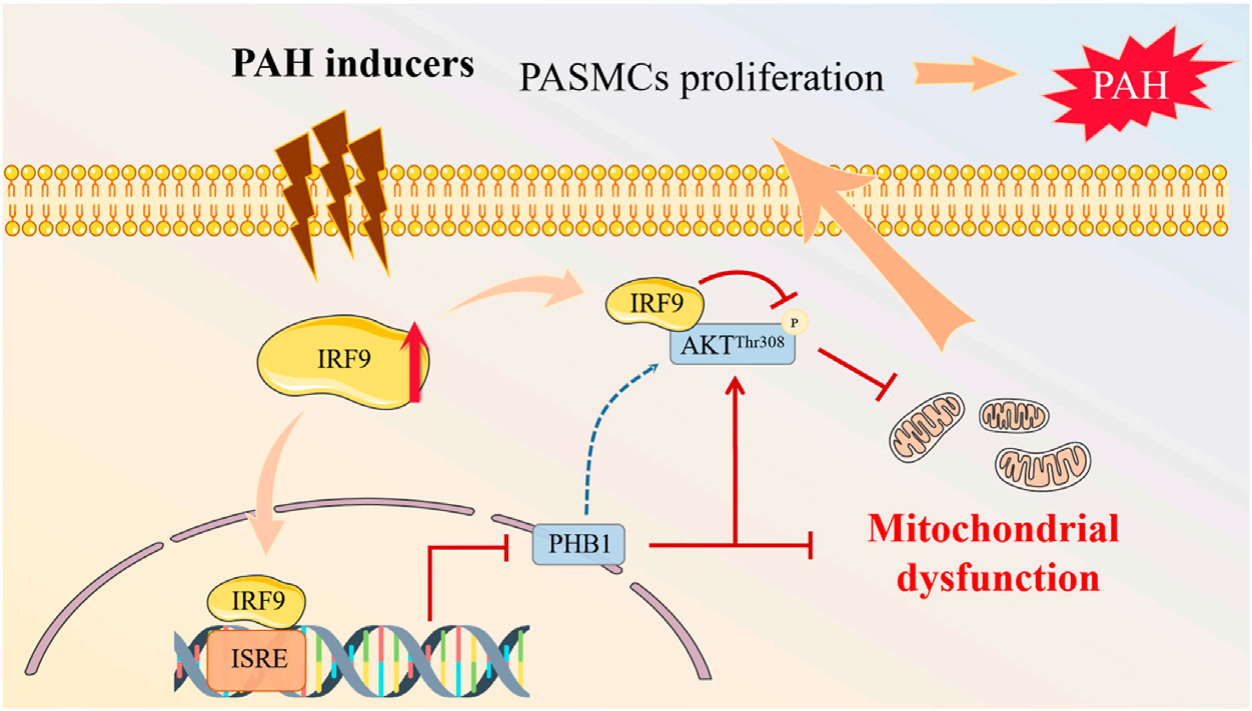
Proposed mechanisms of IRF9 regulates proliferation of PASMCs in PAH. Multiple PAH inducers promote the expression of IRF9. On the one hand, increased IRF9 directly interacts with AKT to regulate the phosphorylation of AKT at T308 site, and on the other hand, IRF9 acts as a transcription factor to inhibit the expression of PHB1, which together leads to mitochondrial dysfunction in PASMCs, which in turn promotes PASMCs proliferation, and ultimately contributes to PAH development.

In summary, we demonstrate that the expression of IRF9 is elevated during PAH development. IRF9 gain-of-function reduces the expression of PHB1 and suppresses the activation of the AKT/GSK3β pathway to promote the proliferation of PASMCs. Our findings shed new light on the role of IRF9 in vascular pathology and implicate a newly identified “IRF9- PHB1-AKT axis” in the pathogenesis of PAH, which provides a new alternative target for the molecular therapy of PAH (Figure 7).

## Data Availability

The original contributions presented in the study are included in the article/Supplementary Materials, further inquiries can be directed to the corresponding authors.
