# Supplementary material for: Upregulation of IRF9 Contributes to Pulmonary Artery Smooth Muscle Cell Proliferation During Pulmonary Arterial Hypertension
Source: Front Pharmacol. 2021 Dec 1;12:773235. doi: 10.3389/fphar.2021.773235 (PMC8672195; doi:10.3389/fphar.2021.773235)
Supplement: Supplementary file 1 [file DataSheet1.pdf]

# Supplemental information

**Upregulation of IRF9 contributes to pulmonary artery smooth muscle cell proliferation during pulmonary arterial hypertension**

Yong-Jie Chen[1,2*], Yi Li[1*], Xian Guo[1], Bo Huo[1], Yue Chen[1], Yi He[1], Rui Xiao[3,4], Xue-Hai Zhu[1,5#], Ding-Sheng Jiang[1,5#], Xiang Wei[1,5#]

[1]Division of Cardiothoracic and Vascular Surgery, Sino-Swiss Heart-Lung Transplantation Institute, Tongji Hospital, Tongji Medical College, Huazhong University of Science and Technology, Wuhan, Hubei, China; [2]Department of Cardiovascular Surgery, Union Hospital, Fujian Medical University, Fuzhou, China; [3]Department of Pathophysiology, School of Basic Medicine, Tongji Medical College, Huazhong University of Science and Technology, Wuhan, China. [4]Key Laboratory of Pulmonary Diseases of Ministry of Health, Tongji Medical College, Huazhong University of Science and Technology, Wuhan, China. [5]Key Laboratory of Organ Transplantation, Ministry of Education; NHC Key Laboratory of Organ Transplantation; Key Laboratory of Organ Transplantation, Chinese Academy of Medical Sciences, Wuhan, Hubei, China.

***These authors contribute equally to this work**

**Correspondence to**

**Xue-Hai Zhu, MD**

Division of Cardiothoracic and Vascular Surgery

Tongji Hospital, Tongji Medical College

Huazhong University of Science and Technology

1095 Jiefang Ave., Wuhan 430030, China

Tel/Fax: 86-27-6937-8454; E-mail: 13072724207@163.com

Or

**Ding-Sheng Jiang, MD**

Division of Cardiothoracic and Vascular Surgery

Tongji Hospital, Tongji Medical College

Huazhong University of Science and Technology

1095 Jiefang Ave., Wuhan 430030, China

Tel/Fax: 86-27-6937-8454; E-mail: jds@hust.edu.cn

Or

**Xiang Wei, MD**

Division of Cardiothoracic and Vascular Surgery

Tongji Hospital, Tongji Medical College

Huazhong University of Science and Technology

1095 Jiefang Ave., Wuhan 430030, China

Tel/Fax: 86-27-6937-8454; E-mail: xiangwei@tjh.tjmu.edu.cn

**Key words:** Pulmonary arterial hypertension; Pulmonary artery smooth muscle cells; Interferon regulatory factor 9; Mitochondrial function; AKT; PHB1

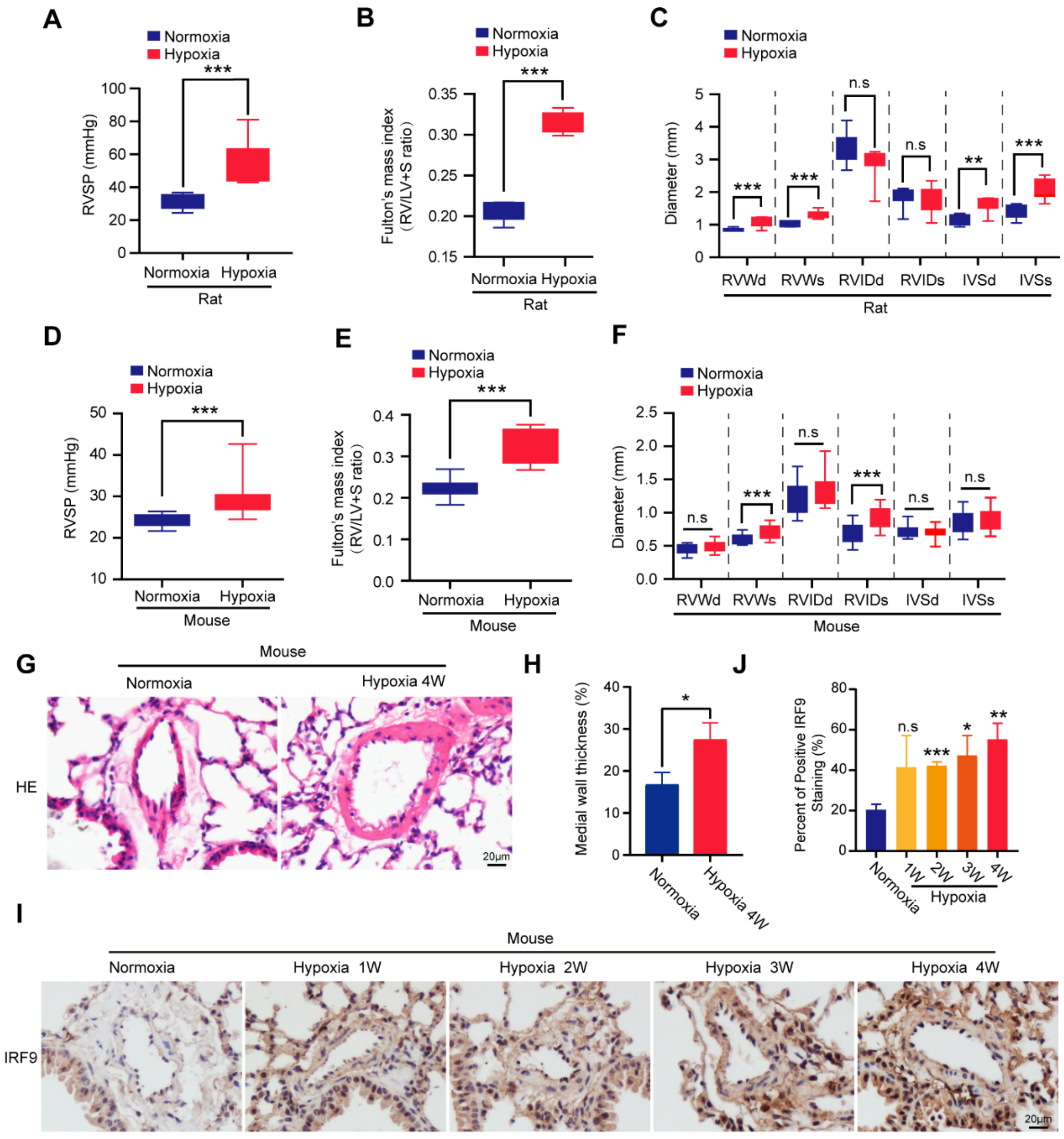


**Supplemental Figure 1. Severe PAH and right heart failure occurred in CH-induced rat and mouse models and IRF9 was upregulated in the CH-induced mouse model.** (**A-C**) In rats, after exposing to normoxia or hypoxia for 4 weeks, right ventricle systolic pressure (RVSP) (A) was measured by right heart catheterization, left ventricle (LV), right ventricle (RV) and interventricular septum (IVS) were sampled and weighed, and Fulton`s mass index was measured (B), and right ventricular wall thickness at end-diastole (RVWd), right ventricular wall thickness at end-systole

(RVWs), right ventricular inside diameter at end-diastole (RVIDd), right ventricular inside diameter at end-systole (RVIDs), interventricular septum thickness at end-diastole (IVSd), interventricular septum thickness at end-systole (IVSs) were assessed by serial echocardiogram (C) (n=8 per group). (**D-F**) In mice exposed to normoxia or hypoxia for 4 weeks, RVSP was measured by right heart catheterization (D); LV, RV and IVS were weighed, and Fulton's mass index was measured (E); RVWd, RVWs, RVIDd, RVIDs, IVSd, IVSs were measured by serial echocardiograms (F) (n=13 for the normoxia group, n=16 for the hypoxia group). (**G**) The representative pulmonary arteriole with Haematoxylin and eosin (HE) staining in the lung sections of control group and 4 weeks chronic hypoxia-induced mice PAH model. Scale bar=20 μm. (**H**) The statistics of medial wall thicknesses [inner diameter/(inner diameter + outside diameter)] of pulmonary arteriole in (G) (n=6 for the normoxia group, n=3 for the hypoxia group). (**I**) The typical pulmonary arteriole with immunohistochemical staining of IRF9 in the lung sections of control group and 1-, 2-, 3- and 4-weeks chronic hypoxia rat PAH model. Scale bar=20 μm. (**J**) According to the results of immunohistochemical staining, the intensity of medial IRF9 staining was quantified relative to the area of the medial layer (n=3 rats per group). Values are means ± SD; ***$p < 0.001$, **$p < 0.01$, *$p < 0.05$, n.s indicates non significance.

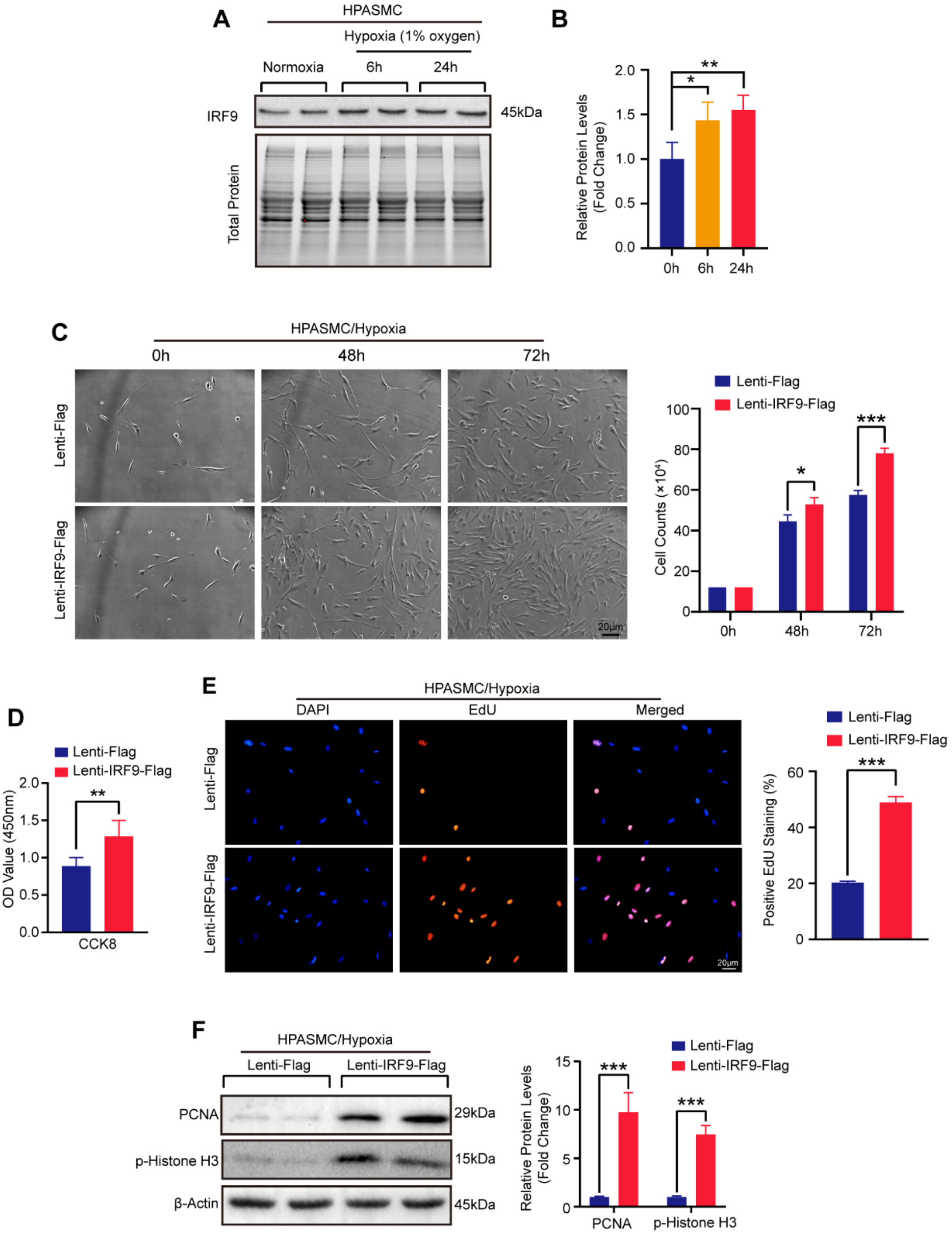


**Supplemental Figure 2. IRF9 overexpression increased the proliferation of HPASMCs under hypoxia condition.** (**A**) After exposing HPASMCs to hypoxia (1% oxygen concentration) for 0 h, 6 h and 24 h, IRF9 protein levels were verified by western blot analysis. (**B**) The protein level was normalized to total protein and

quantified (n=3 independent experiments). (**C**) Representative images show the density of IRF9-overexpressing (Lenti-IRF9-Flag) HPASMCs and the control group (Lenti-Flag) culturing for 0 h, 48 h and 72 h in 1% oxygen concentration (left panel). Scale bar, 20 μm. Cell numbers were counted at each time point (right panel) (n=3 independent experiments). (**D**) CCK-8 assay was performed and the absorbance (OD value) at 450 nm was measured to shows the proliferation activity of Lenti-Flag and Lenti-IRF9-Flag HPASMCs under hypoxia condition (n=3 independent experiments). (**E**) Typical images of EdU assay show the ratio of EdU incorporated HPASMCs of Lenti-Flag and Lenti-IRF9-Flag groups under hypoxia condition. Nuclei were stained with DAPI (blue) and EdU incorporation appear in red (left panel). The positive EdU staining rate was measured (right panel) (n=3 independent experiments). Scale bar, 20 μm. (**F**) The protein levels of proliferation biomarker PCNA and p-Histone H3 in Lenti-Flag and Lenti-IRF9-Flag HPASMCs were determined using western blot under hypoxia condition (left panel). The expression levels were normalized to β-Actin and quantified (right panel) (n=3 independent experiments). (**D**-**F**) After infection, HPASMCs were culturing in hypoxia condition (1% oxygen concentration) for 48h and then sampled for indicated experiments. Values are means ± SD; $^{***}p < 0.001$, $^{**}p < 0.01$, $^{*}p < 0.05$.

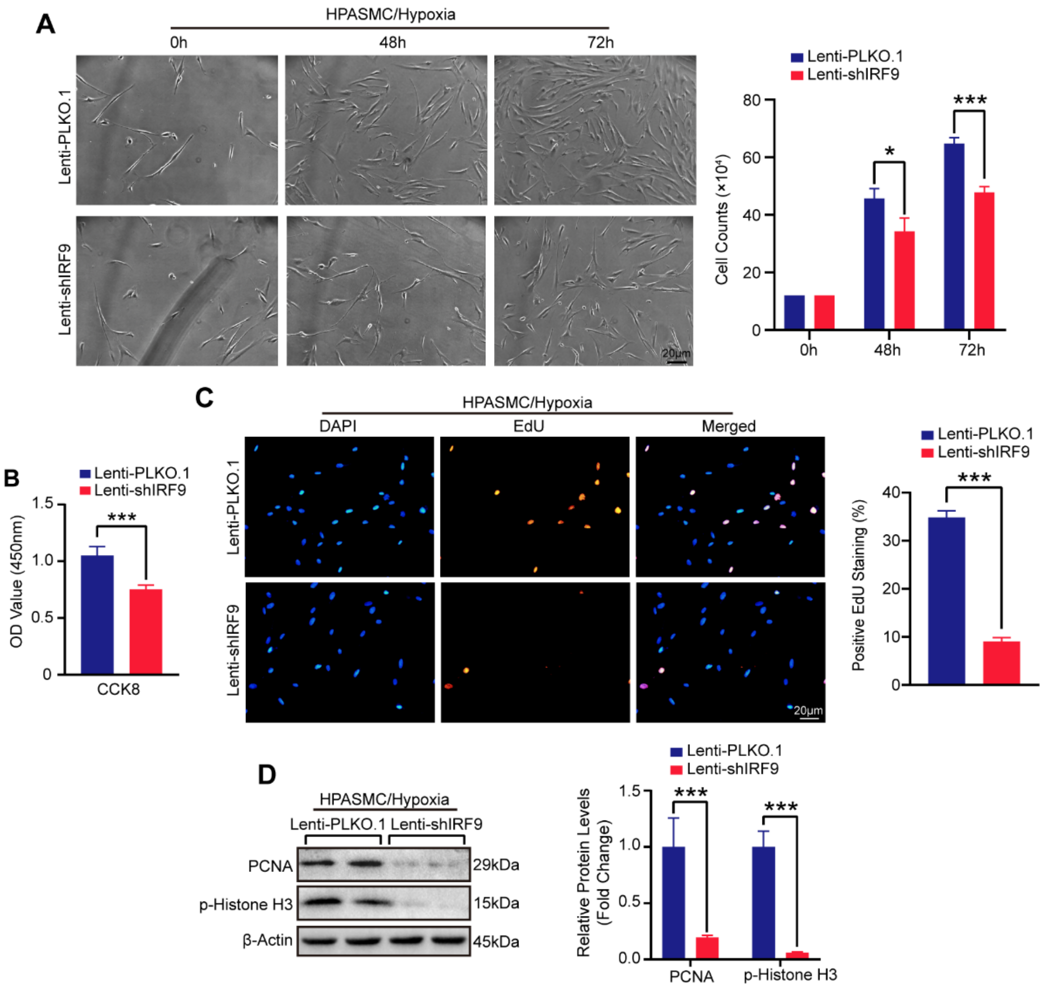


**Supplemental Figure 3. IRF9 knockdown decrease the proliferation of HPASMCs under hypoxia condition.** (A) Representative images show the density of HPASMCs with IRF9-knockdown (Lenti-shIRF9) or not (Lenti-PLKO.1) after culturing for 0 h, 48 h and 72 h under hypoxia (1% oxygen concentration) condition (left panel). Scale bar, 20 μm. Cell numbers were counted at each time point (right panel) (n=3 independent experiments). (B) CCK-8 assay shows the decrease of absorbance at 450 nm which indicate the attenuation of proliferation capacity in Lenti-shIRF9 HPASMCs comparing to Lenti-PLKO.1 under hypoxia condition (n=3 independent experiments). (C) Typical images show the ratio of EdU incorporation in Lenti-PLKO.1 and Lenti-shIRF9 HPASMCs under hypoxia condition, in which nuclei were stained with DAPI

(blue) and EdU incorporation appear in red (left panel). Scale bar, 20 μm. The positive EdU staining rate was measured (right panel) (n=3 independent experiments). (**D**) Protein levels of proliferation biomarkers PCNA and p-Histone H3 were determined by western blot in Lenti-PLKO.1 and Lenti-shIRF9 HPASMCs (left panel). The protein levels were normalized to β-Actin and quantified (right panel) (n=3 independent experiments). (**B**-**D**) After infection, HPASMCs were culturing in hypoxia condition (1% oxygen concentration) for 48h and then sampled for indicated experiments. Values are means ± SD; ***p < 0.001, *p < 0.05.

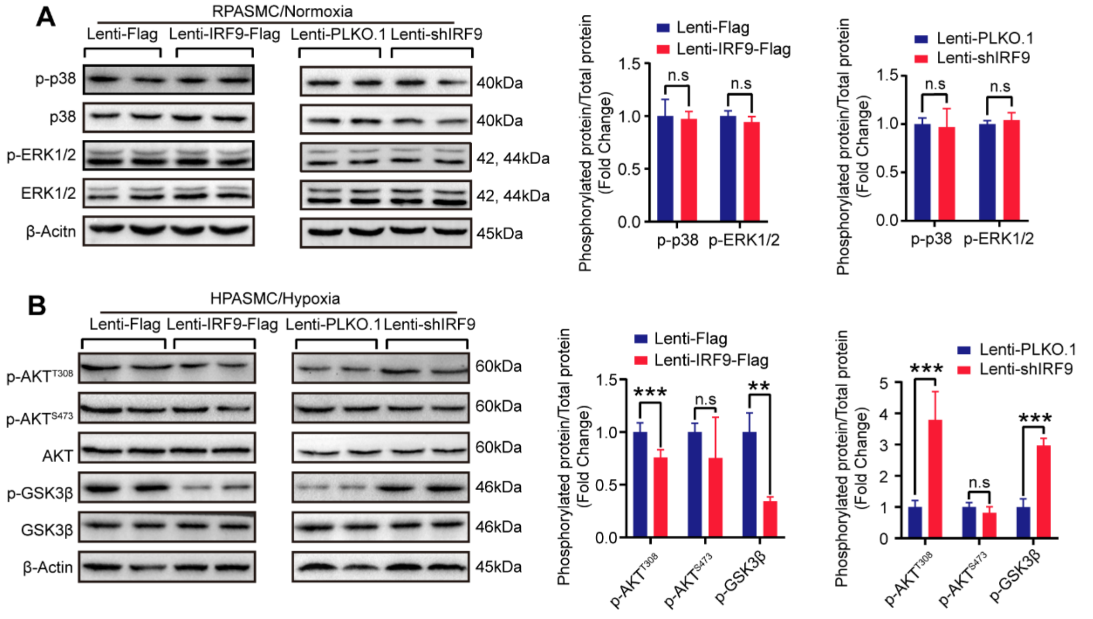


**Supplemental Figure 4. IRF9 regulates the PASMCs proliferation through the AKT/GSK3β pathway but not p-38, ERK 1/2 under hypoxia condition.** (**A**) Representative western blots (left panel) and quantification results (right panel) of p-p38, p38, p-ERK1/2, ERK1/2 in IRF9-overexpressing (Lenti-IRF9-Flag), IRF9-knockdown (Lenti-shIRF9) and the control group (Lenti-Flag/Lenti-PLKO.1) of RPASMCs under normoxia condition (left panel). The relative levels of phosphorylated proteins were normalized by their total protein respectively (n=3 independent experiments). β-Actin serves as loading control. (n=3 independent experiments). (**B**) representative western blots (left panel) and quantification results (right panel) of p-AKT$^{T308}$, p-AKT$^{S473}$, AKT, p-GSK3β and GSK3β in IRF9-overexpressed (Lenti-IRF9-Flag) or knocked down (Lenti-shIRF9) HPASMCs under hypoxia condition. The relative levels of phosphorylated proteins were normalized by their total protein respectively (n=3 independent experiments). β-Actin serves as loading control. Values are means ± SD; ***$p < 0.001$, **$p < 0.01$, n.s indicates non significance.

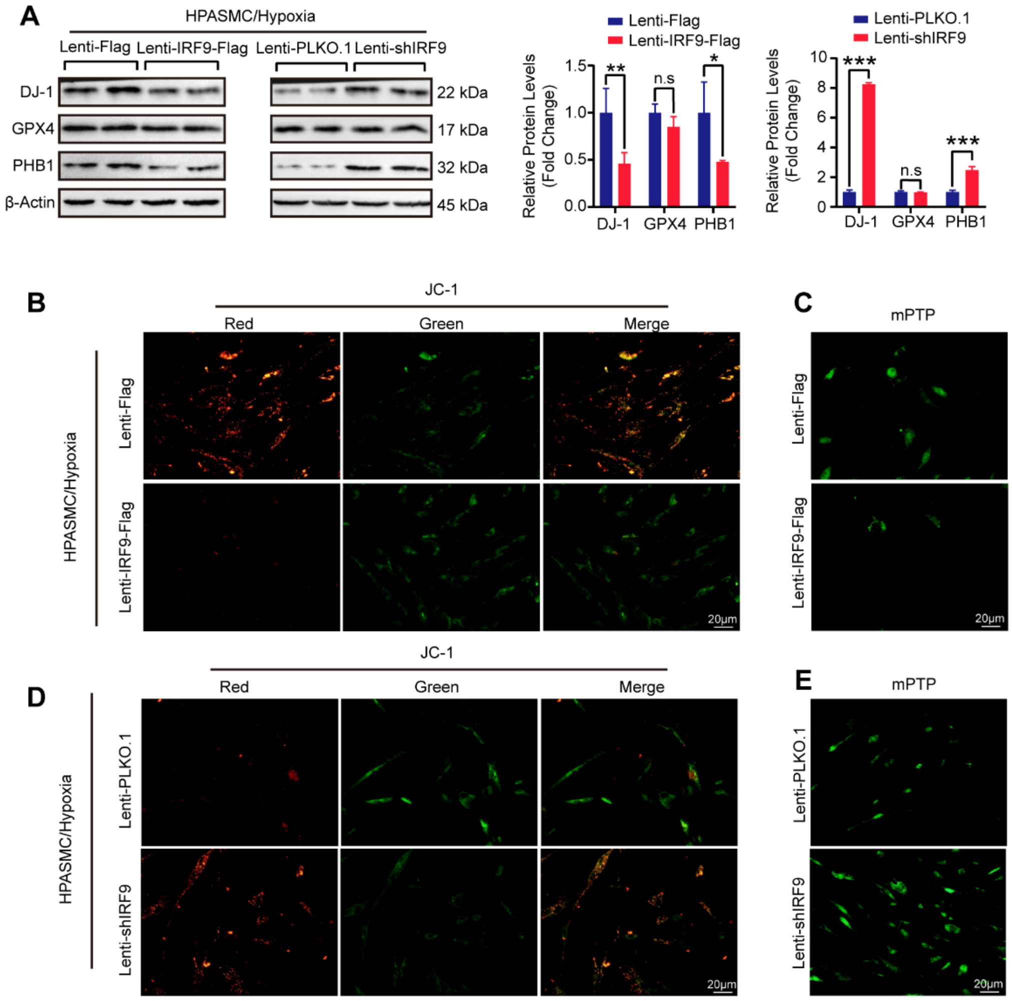


**Supplementary Figure 5. IRF9 affects the mitochondria function through regulating PHB1 expression under hypoxia condition.** (**A**) The protein levels of DJ-1, GPX4 and PHB1 in IRF9-overexpressing (Lenti-IRF9-Flag), IRF9-knockdown (Lenti-shIRF9) and neither (Lenti-Flag/Lenti-PLKO.1) HPASMCs were assessed by western blotting (left panels) under hypoxia condition. Proteins levels were quantified and normalized to β-Actin (right panels) (n=3 independent experiments). (**B**, **D**) The representative images of mitochondrial membrane potential detected by using JC-1 kit in HPASMCs with IRF9 overexpression (B) or knockdown (D) under hypoxia condition. Scale bar, 20 μm (n=3 independent experiments). (**C**, **E**) The openness of

mitochondrial permeability transition pore (mPTP) is detected by the mPTP kit in HPASMCs with IRF9 overexpression (C) or knockdown (E) under hypoxia condition, Scale bar, 20 μm (n=3 independent experiments). (**A-E**) After infection, HPASMCs were culturing in hypoxia condition (1% oxygen concentration) for 48h and then sampled for indicated experiments. Values are means ± SD; ***p < 0.001, **p < 0.01, n.s indicates non significance.
